# Prevalence of Intracranial and Cervical Artery Abnormalities in Patients with Hypermobile Ehlers–Danlos Syndrome and Hypermobility Spectrum Disorders Presenting to an Academic Headache Clinic

**DOI:** 10.3390/neurolint18020033

**Published:** 2026-02-11

**Authors:** Todd D. Rozen, Katelyn A. Bruno, Ethan M. Rozen, Frances C. Wilson, Marysia S. Tweet, Raymond C. Shields, Sharonne N. Hayes, Dacre R. T. Knight, Shilpa N. Gajarawala, Sukhwinder J. S. Sandhu, Alok A. Bhatt, DeLisa Fairweather

**Affiliations:** 1 Division of Neurology, Mayo Clinic, Jacksonville, FL 32224, USA; 2 Division of Cardiovascular Medicine, Department of Medicine, University of Florida, Gainesville, FL 32611, USA; 3 Department of Cardiovascular Medicine, Mayo Clinic, Jacksonville, FL 32224, USA; 4 Department of General Internal Medicine, Mayo Clinic, Jacksonville, FL 32224, USA; 5 Department of Cardiovascular Medicine, Mayo Clinic, Rochester, MN 55905, USA; 6 Division of Neuroradiology, Mayo Clinic, Jacksonville, FL 32224, USA; sandhu.johnny@mayo.edu (S.J.S.S.);

**Keywords:** hypermobile Ehlers–Danlos syndrome, hypermobility spectrum disorders, intracranial aneurysm, cervical artery dissection, fibromuscular dysplasia, migraine

## Abstract

**Background/Objective:** It remains unknown whether patients with the more common forms of hypermobility carry an elevated risk for the development of intracranial/cervical artery abnormalities. The objective of this study was to determine the prevalence of unruptured intracranial aneurysms, spontaneous cervical artery dissections, and fibromuscular dysplasia in patients with hypermobile Ehlers–Danlos Syndrome (hEDS) and hypermobility spectrum disorders (HSD) who presented to an academic headache clinic. **Methods:** This is a retrospective cohort study. We used an electronic medical record to look for all patients seen at the Mayo Clinic Florida Headache Center and EDS Clinic between 2019 and 2025 with a diagnosis of hEDS or HSD and neuroimaging of both the intracranial and cervical arteries. **Results:** There were 103 patients who met the inclusion criteria. There was no statistically significant difference between hEDS and HSD patients in developing cerebral/cervical arterial anomalies. Of the sample, 95% of the hypermobile patients with abnormal neuroimaging also had migraine. A total of eleven (10.7%) patients (hEDS + HSD) were diagnosed with unruptured intracranial aneurysms. Trends included age less than 50 years, small aneurysms in the anterior circulation, and having migraine with aura. Five (4.8%) patients were diagnosed with spontaneous cervical artery dissection with trends for HSD, over the age of 50 years, vertebral artery involvement and a history of migraine without aura. Six (5.8%) patients were diagnosed with fibromuscular dysplasia with trends for HSD, over the age of 50 years, carotid artery involvement and a history of migraine with aura. **Conclusions:** This is the first study to identify that patients with the more common type of EDS, HSD and hEDS, and a possible concomitant history of migraine have a heightened risk for the development of unruptured intracranial aneurysms, spontaneous cervical artery dissections, and fibromuscular dysplasia. Our findings suggest the need for targeted screening with intracranial and extracranial arterial imaging for this unique patient population.

## 1. Introduction

Hypermobile Ehlers–Danlos syndrome (hEDS) and hypermobility spectrum disorders (HSD) are the most common forms of hypermobility, estimated to affect upwards of 250 million individuals globally [1]. They are both female predominant conditions with a reported sex ratio of at least 9:1, female to male [2,3]. They appear to be heritable, but no recognized genetic mutation has been found for either disorder, and the true underlying pathogenesis of each condition has yet to be established. Even though hEDS and HSD can present with similar physical characteristics and some of the same multi-system medical issues, they display some unique differences in the presentation of symptoms and comorbidities [4]. Connective tissue disorders with the worst outcomes, including Marfan syndrome and vascular EDS (vEDS), have a higher prevalence of cerebral and cervical arterial abnormalities than the general population [5]. However, there is a lack of large studies investigating the prevalence of these issues in hEDS/HSD patients, with only case studies in the published literature [5]. What is unknown at present is whether the more common forms of hypermobility (hEDS and HSD) are associated with a higher prevalence of arteriopathy similar to vEDS. Additionally, at present, there are no screening neuroimaging guidelines for cerebral and cervical arterial abnormalities in hEDS and HSD populations. We recently examined the prevalence of cardiac issues (aortic root dilation and mitral valve prolapse) in patients with hEDS and HSD using the 2017 diagnostic criteria for EDS [6] and found that approximately 3% of patients had those conditions [7].

Migraine is a primary headache disorder affecting over 1 billion individuals worldwide, with a prevalence of about 15% [8]. There have been some studies suggesting that migraine headache alone could be a risk factor for the development of cerebral/cervical vascular abnormalities, although the exact connection is unknown. Additionally, since migraine is so common, there is no consensus as to whether the observed association is a true risk factor [9,10,11]. To make this issue even more complicated, patients with HSD or hEDS appear to have a higher rate of developing migraine, both episodic and chronic forms, than the general population [3,12]. Thus, the question arises in patients with a migraine history who develop neurovascular anomalies: What percentage of those patients are also hypermobile, and does hypermobility alone, migraine alone, or both constitute a true risk factor?

We present a retrospective cohort study examining individuals who first presented to an academic headache clinic with a complaint of head pain, who demonstrated hypermobility issues based on history and examination, and who were then seen at the Mayo Clinic Florida EDS Clinic for a specific diagnosis. Headache patients diagnosed with hEDS or HSD were imaged to screen for the presence of cerebral/cervical arterial issues, including unruptured intracranial aneurysms (UIA), spontaneous cervical artery dissections (SCeAD), and fibromuscular dysplasia (FMD). The goal of the study was to determine the prevalence of these vessel abnormalities in patients with hEDS and HSD who suffer from headache or migraine, and to determine whether targeted neuroimaging screening is suggested for this specific patient cohort. Finally, we discuss whether hypermobility alone, migraine alone, or a combination of both may be risk factors for the development of cerebral/cervical arterial abnormalities.

## 2. Materials and Methods

### 2.1. Ethics Statement

The Institutional Review Board (IRB# 19-011260, date 29 May 2025) of the Mayo Clinic approved the retrospective analysis of demographic and clinical data from medical records for this study and waived informed consent for all patients. The research conformed to the principles outlined in the Declaration of Helsinki.

### 2.2. Study Design

**Figure 1:**
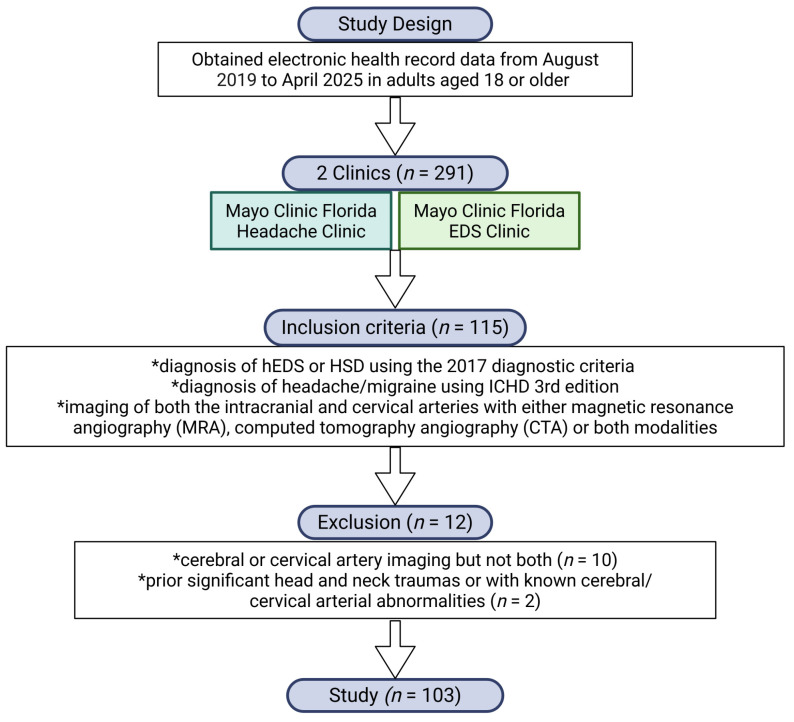
Inclusion and exclusion criteria for the study. Created in BioRender. Fairweather, D. (2026) https://BioRender.com/ih2d9rt (accessed on 1 February 2026).

We used the Mayo Clinic electronic medical record to identify patients seen by a headache fellowship-trained neurologist (TDR), between August 2019 and April 2025 at the Mayo Clinic Florida Headache Center, and who also attended the Mayo Clinic EDS Clinic [13]. All patients were adults 18 years of age or older. In total, 291 patients met those criteria. Of those, 115 patients were diagnosed with HSD or hEDS and had specific neuroimaging. Those with only cerebral or cervical artery imaging but not both were excluded from the study (*n* = 10). Those with prior significant head and neck traumas or with known cerebral/cervical arterial abnormalities were also excluded (*n* = 2). After those exclusions, 103 patients were identified with a diagnosis of hEDS or HSD using the 2017 diagnostic criteria [6] that had imaging of both the intracranial and cervical arteries with either magnetic resonance angiography (MRA), computed tomography angiography (CTA), or both modalities (Figure 1).

### 2.3. Patients

The criteria for a diagnosis of hEDS include the identification of generalized joint hypermobility of specific joints using the Beighton Scale, evidence of a systemic connective tissue disorder, family history and/or musculoskeletal complications, and several exclusions [6]. The Beighton Scale assesses the joint hypermobility of select joints, such as knees and elbows [14]. A Beighton Scale score of ≥5/9 after puberty or ≥4/9 after age 50 is a positive score [6]. Patients are diagnosed with HSD if they do not meet the diagnostic criteria for hEDS, have a positive Beighton Scale score, and have evidence that joint hypermobility is causing problems and it is not just an asymptomatic feature (feature C of the 2nd EDS criterion) [6,14]. Patients with localized HSD (Beighton Score > 0 but not meeting criteria for hEDS and hypermobility elsewhere) and those with historical HSD (Beighton Score = 0; hypermobility elsewhere) were not included in this study [15].

### 2.4. Data Collection

The International Classification of Headache Disorders (ICHD) 3rd edition was utilized to diagnose primary headache disorders [16]. A diagnosis of hEDS or HSD was verified for each patient by EDS specialists (DRTK, SG) at the Mayo Clinic EDS Clinic according to the 2017 diagnostic criteria [4,6,16]. We used specific ICD codes to identify positive imaging results for cerebral/intracranial aneurysms (cerebral aneurysm, non-ruptured 167.1, cerebral aneurysm, ruptured 160.8), cervical artery dissection (dissection of unspecified artery 177.70, dissection of carotid artery 177.71, dissection of vertebral artery 177.74), and FMD (arterial, fibromuscular 177.3). All imaging was interpreted by academic neuro-radiologists. All imaging results were verified by a trained neurologist (TDR) for positive and negative findings to identify any errors or any missing or incorrect ICD codes. Five charts were not coded correctly.

### 2.5. Imaging

1. The diagnosis of spontaneous cervical artery dissection was made on the basis of the consensus paper from several academic societies that was published in the Journal of the American College of Cardiology [17]. Dissections can occur spontaneously or even from minor trauma. It results from an intimal tear and subsequent intramural hematoma. Depending on its subintimal or subadventitial location, these result in luminal stenosis or aneurysmal degeneration. In current practice, diagnosis is made by noninvasive imaging, usually CTA or MRA. Luminal abnormalities are the hallmark imaging features and can include a combination of intimal flap, dual lumen, dissecting aneurysm, stenosis, or occlusion. MRI/MRA is particularly useful in identifying luminal hematomas with a characteristic hyperintense T1 signal. CT angiography offers high spatial resolution for the evaluation of an intimal flap. A cervical artery dissection-associated pseudoaneurysm occurs when there is a hematoma contained by the adventitial layer of the vessel, with imaging demonstrating irregularity of the vessel wall with focal outpouching [18].
2. Fibromuscular dysplasia diagnoses were established with non-invasive imaging with CTA, which has supplanted the previous methods of catheter-based cerebral angiography. The imaging findings are characteristic of alternating areas of stenosis and dilatation of the mid-to-distal cervical internal artery in a classic “string-of-pearls” or beaded pattern. S-curve deformities and tortuosity of the vessel can also be seen. Absence of plaque at the common carotid artery bifurcation is also useful in distinguishing from typical atherosclerotic disease [19].

### 2.6. Statistical Analysis

Continuous variables were summarized with the sample median and range. Categorical variables were summarized with the number and percentage of subjects. Welch’s *t*-test was used to compare the differences in continuous variables among groups. A Fisher’s exact test examined the association between two categorical variables. All the tests were two-tailed, and *p* values <0.05 were considered statistically significant. All statistical analyses were performed using Excel or GraphPad Prism. Graphs/images were created using GraphPad Prism (Version 10.3.1) and BioRender. Because of our overall small numbers for positive vessel abnormalities on imaging and a small total population cohort, we could not do a full statistical analysis on most variables, but rather suggest trends.

## 3. Results

### 3.1. Demographics

We identified 103 patients who met our inclusion criteria of having attended the Mayo Clinic Headache and EDS Clinics, received a diagnosis of HSD or hEDS, received a diagnosis of a headache or migraine, and had imaging of both cerebral and cervical arteries. The demographics of the patient cohort are listed in Table 1.

An HSD diagnosis was made in 72 (69.9%) and hEDS in 31 (30.1%) patients. Overall, there were 99 females (96.1%) diagnosed with HSD or hEDS and four males (3.9%) (*p* = 0.99), a sex ratio of 24.7:1, female to male. Patients diagnosed with HSD had a mean age of 37 years (range 18–67) while patients with hEDS had a mean age of 38 years (range 20–55) (*p* = 0.97). HSD was diagnosed in 69 women (95.8%) and 30 with hEDS (96.8%). Three men had HSD (4.2%), while one was diagnosed with hEDS (3.2%). Both HSD and hEDS patients overwhelmingly identified as White and of non-Hispanic ethnicity, with low numbers of Black/African Americans and Asians, with no differences by diagnosis (White *p* = 0.10, non-Hispanic *p* = 0.22). Thus, the demographics of HSD and hEDS patients with headache/migraine were similar (Table 1).

Overall, 91 of 103 (88.3%) patients with HSD or hEDS had a history of migraine, 54 (52.4%) were diagnosed with episodic migraine, 37 (35.9%) had chronic migraine, and 12 (11.6%) had new daily persistent headache (NDPH) (Table 1). For HSD patients, 63 (87.5%) had migraine, 37 (51.4%) had episodic migraine, 26 (36.1%) had chronic migraine, and nine (12.5%) had NDPH (Table 1). Similarly, for hEDS patients, 28 (90.3%) had migraine, 17 (54.8%) had episodic migraine, 11 (35.5%) had chronic migraine, and three (9.7%) had NDPH (Table 1). Overall, in females, 88 had migraine, 52 had episodic migraine (35 HSD, 17 hEDS), 36 had chronic migraine (26 HSD, 10 hEDS), and 11 had NDPH (seven HSD, four hEDS). In males, three had migraine, two had episodic migraine (both with HSD), one had chronic migraine (hEDS), and one had NDPH (HSD). Episodic migraine was about 1.5 times more likely to occur than chronic migraine for both hEDS and HSD populations. Overall, there were no significant differences between HSD and hEDS diagnoses for migraine types (Table 1). In our study cohort, no other primary headache diagnoses were noted outside of migraine and NDPH. In regard to secondary headaches, none of our patients met ICHD-3 criteria for headache attributed to cranial or cervical vascular disorders [16]. This was based on the headache history starting years prior to positive imaging results and no acute/subacute changes in headache, the lack of side-locked head pain or thunderclap headache onset, and no abnormal neurologic symptoms or signs associated with headaches. In addition, all patients were ruled out for spontaneous intracranial hypotension, which is associated with hypermobility, by headache history (lack of orthostatic headache), imaging (all brain MRIs included gadolinium looking for pachymeningeal enhancement and brain sag), as well as by exam (negative Trendelenburg test).

### 3.2. Unruptured Intracranial Aneurysm

Because the demographics were similar between HSD and hEDS (Table 1), we determined the prevalence of UIAs in patients diagnosed with HSD and hEDS combined. A total of 11 patients, or 10.7%, with HSD/hEDS and headache/migraine had evidence of UIAs (Table 2).

The overall demographics for patients with HSD or hEDS and UIA were as follows: nine patients were women, two were men, and one patient had two aneurysms (thus, 11 patients with 12 aneurysms overall). According to hypermobility diagnosis, six HSD patients had UIAs (five females, one male), and five hEDS patients (four females, one male) had UIAs, for a 1.2:1 ratio HSD to hEDS. There was no statistically significant difference for developing UIA between hypermobile diagnoses (*p* = 0.30) (Table 2). The average age at UIA diagnosis in females was 41 years (HSD 40 years, hEDS 42 years), and for males it was 30 years of age. Thus, 10 of 11 HSD/hEDS patients were <50 years of age at the time of UIA diagnosis. Nine out of the 12 UIAs were documented at 2–3 mm in size, while three were larger at 6 mm, 7 mm, and 9 mm, with the largest having surgical intervention (mean, 3.8 mm; median, 3 mm). Aneurysm morphology was saccular in nine patients (five HSD, four hEDS) and fusiform in two patients (one HSD, one hEDS). There were no aneurysmal ruptures.

**Figure 2:**
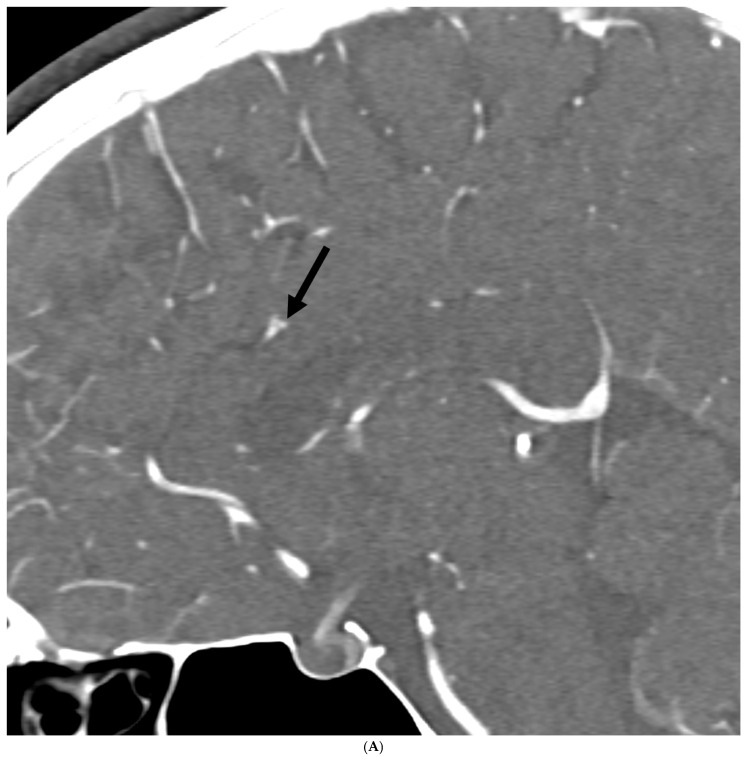

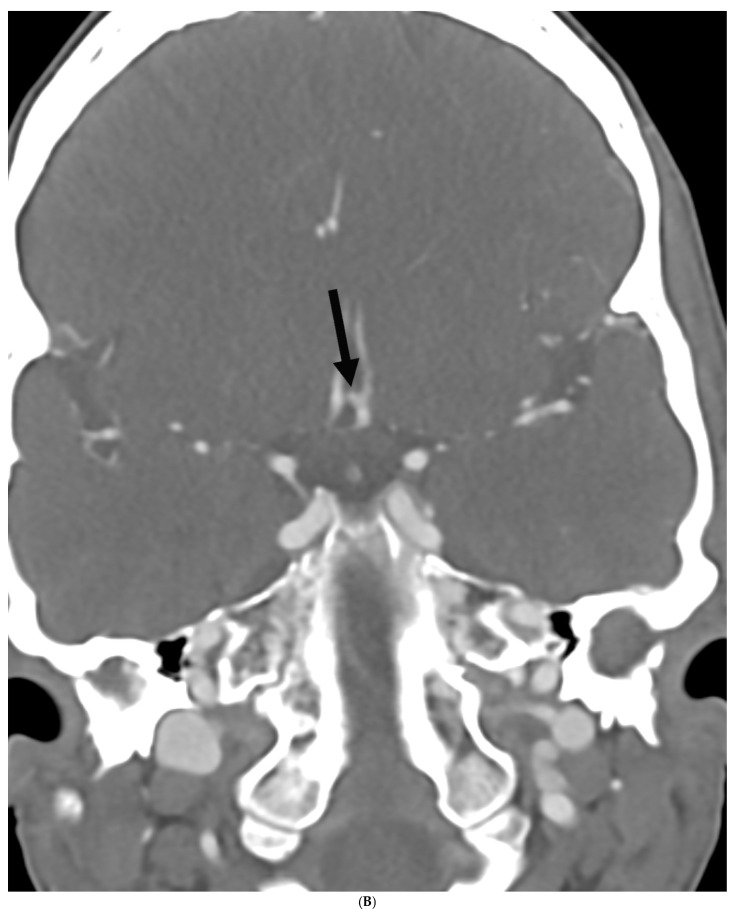
(**A**) 24-year-old woman with hEDS and a history of chronic migraine. Sagittal CT angiography image demonstrates a 2 mm saccular aneurysm from the left pericallosal artery (arrow). (**B**) 36-year-old woman with HSD and a history of chronic migraine. Coronal CT angiography image demonstrates a 2 mm left A1/A2 junction aneurysm (arrow).

Regarding UIA location in hEDS patients, lesions were noted in the left pericallosal artery, left ophthalmic, left petrous carotid, left cavernous carotid, right superior hypophyseal artery, and right petrous carotid. In HSD patients, UIA lesions were located in the left anterior communicating, left cavernous carotid, left A1/A2 junction, left posterior communicating, right cavernous and right pericallosal arteries. Thus, most aneurysms involved the anterior/carotid artery circulation (10/11 patients and 11/12 aneurysms). None of the patients had a prior smoking history. Only one patient (hEDS) had a possible family history of intracranial aneurysms in a parent, but prior records could not be obtained to define the etiology of an intracranial hemorrhage. Figure 2A,B are representative images of UIA in HSD and hEDS patients.

Regarding headache diagnoses in patients with UIAs, 10 of 11 HSD/hEDS patients had migraine (six chronic migraine, four episodic migraine, six with a history of migraine with aura), and one patient had NDPH. For HSD patients, four had episodic migraine, and two had chronic migraine; while for hEDS patients, four had chronic migraine and one had NDPH. Thus, the majority of HSD patients with UIAs had episodic migraine, whereas a majority of hEDS patients had chronic migraine. Slightly more than 50% of hypermobile patients with UIA also had a history of migraine with aura (three with HSD and three with hEDS).

### 3.3. Spontaneous Cervical Artery Dissection

**Figure 3:**
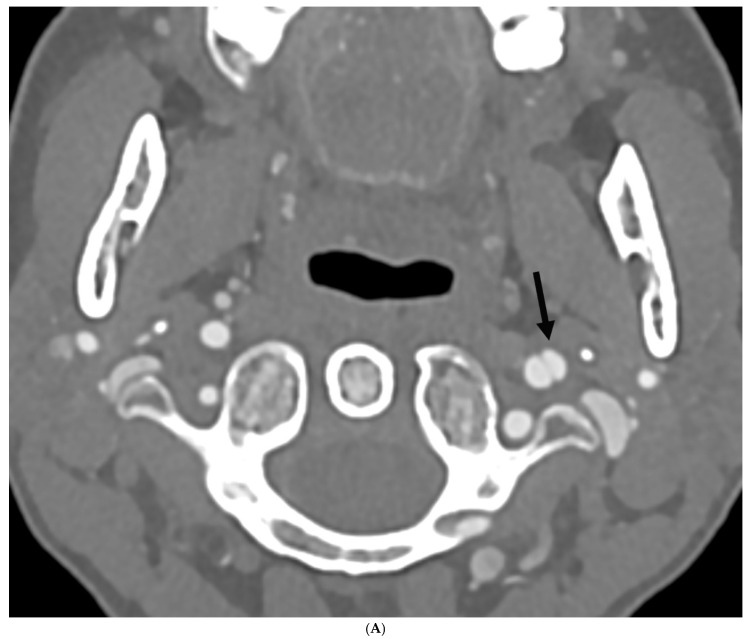

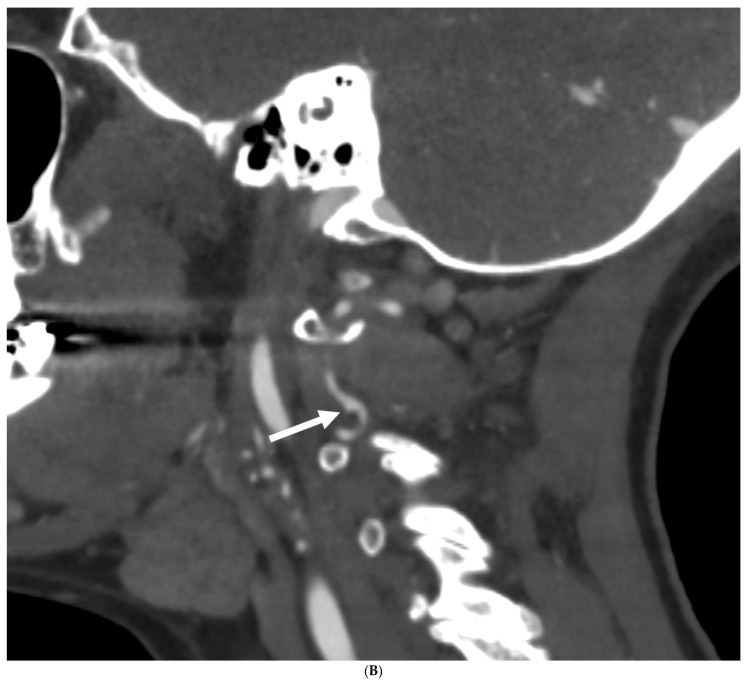
(**A**) 67-year-old woman with hEDS and a history of chronic migraine. Axial CT angiography image demonstrates a laterally projecting pseudoaneurysm of the left mid cervical internal carotid artery measuring up to 2.6 mm in depth (arrow), a sequelae of prior dissection. (**B**) 60-year-old woman with HSD and a history of episodic migraine. Sagittal CTA image demonstrates a tiny focal outpouching of the V3 segment of the left vertebral artery (arrow) compatible with a tiny pseudoaneurysm, a sequelae of prior dissection.

A total of five patients, or 4.8%, with hEDS and HSD and headache/migraine were diagnosed with SCeAD (Table 2). The demographics and location of the lesion in HSD/hEDS patients with SCeAD were as follows: four of five were female, four had HSD (three female, one male), and one had hEDS (female) (Table 2). There was no statistically significant difference for developing SCeAD between hypermobile diagnoses (*p* = 1.00) (Table 2). The mean average age at time of SCeAD diagnosis for females was 51 years (range 24–66 years), with three of four women being ≥55 years of age. The male patient was 54 years old at the time of SCeAD diagnosis. Location of SCeAD in our population noted three of the five patients had vertebral artery dissections involving the left vertebral artery, while one patient also had a right vertebral artery dissection (bilateral dissections). All female patients with HSD had vertebral artery dissections, while the one male with HSD had bilateral carotid artery dissections. The one female patient with hEDS developed left carotid dissection. Two of the women (one HSD, one hEDS) had concomitant mild FMD that involved the contralateral side to their dissection, suggesting against a causative effect. Only one of the five patients (female, with HSD) had a prior smoking history. Figure 3A,B are representative images of SCeAD in HSD and hEDS patients.

All patients with dissections had a history of migraine. In females with HSD, two had episodic migraine (one with aura), one had chronic migraine (without aura), while the hEDS patient had chronic migraine with aura. The sole male patient with HSD had episodic migraine without aura. None of the patients developed an ischemic/neurologic event, and none required surgical intervention.

### 3.4. Fibromuscular Dysplasia

**Figure 4:**
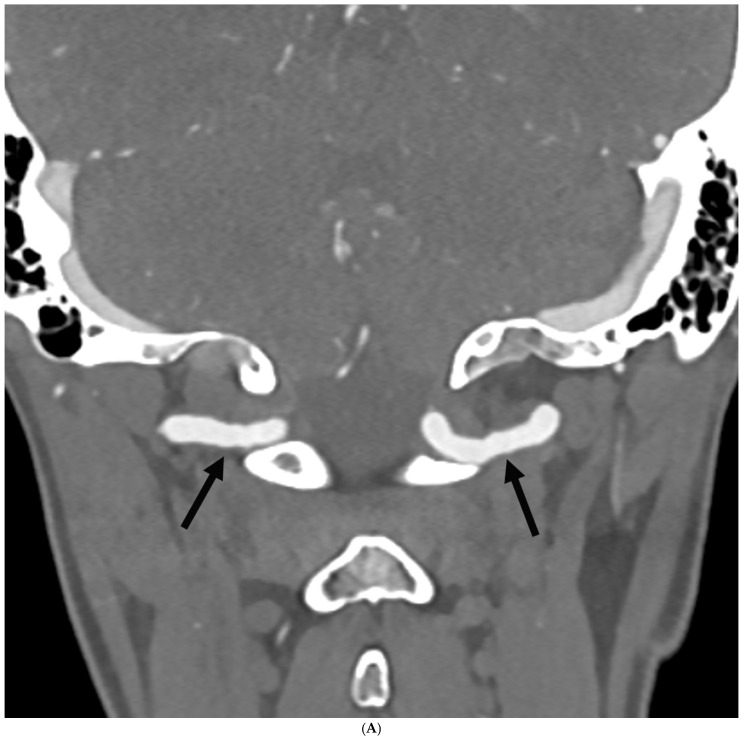

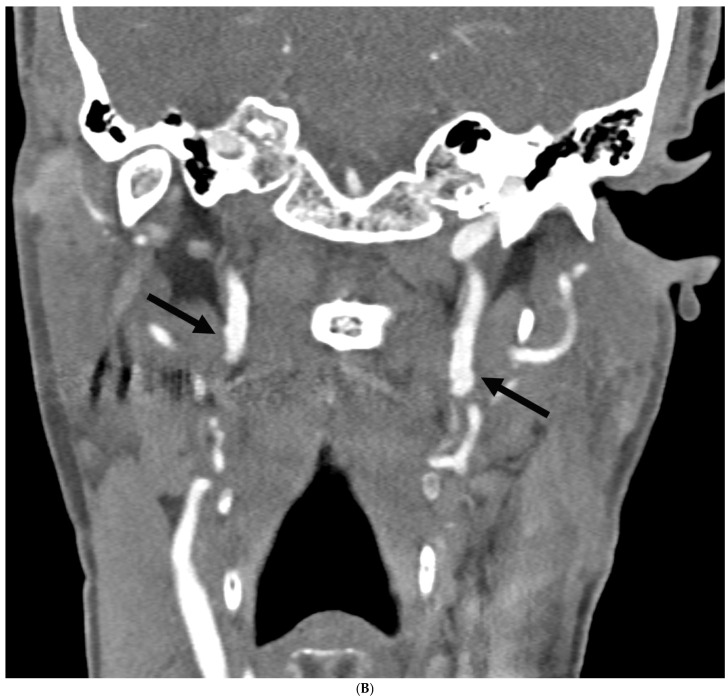
(**A**) 65-year-old woman with hEDS and a history of chronic migraine. Coronal CT angiography image demonstrates mild luminal irregularity (“beaded appearance”) of the V3 segments of the bilateral vertebral arteries (arrows), compatible with FMD. (**B**) 53-year-old woman with HSD and a history of episodic migraine. Coronal CT angiography image demonstrates a beaded appearance of the bilateral cervical internal carotid arteries (arrows), compatible with FMD.

A total of six patients, or 5.8%, with HSD/hEDS and headache/migraine had FMD identified in the head and neck vessels. (Table 2). All HSD/hEDS patients with FMD were women, and five out of six had HSD (Table 2). Five of six patients were diagnosed at ≥50 years of age, while one patient was 29 years of age. The mean age at diagnosis was 54 years. There was no statistically significant difference in developing FMD between hypermobile diagnoses (*p* = 0.67) (Table 2). In regard to imaging modalities utilized, in four patients MRA suggested FMD and was verified by CTA, while in two patients, CTA was the initial test and identified the FMD. FMD involved the carotid arteries in all patients, while the one patient with hEDS also had vertebral artery involvement. Two patients had bilateral artery involvement. In four patients (all with HSD), the FMD lesions were focal, while in two patients (one hEDS, one HSD), they were multifocal. FMD was graded as mild in all patients without any significant artery stenosis. None of the patients had a prior smoking history. Figure 4A,B are representative images of FMD in HSD and hEDS patients.

Regarding headache diagnoses in FMD patients, all patients had a history of migraine. Two HSD patients had episodic migraine, while four patients had chronic migraine (three HSD, one hEDS). Five of the six patients (four HSD, one hEDS) also had a migraine with aura history.

## 4. Discussion

One goal of this study was to determine the prevalence of cerebral/cervical arterial vessel abnormalities in patients with HSD and hEDS who also suffered from headache or migraine and were cared for at a tertiary referral center. Because the demographics of the patients were similar (Table 1), we combined patients diagnosed with HSD and hEDS in the prevalence estimate. In HSD/hEDS patients with headache/migraine in this study, we found that 10.7% had UIAs, 4.8% had SCeAD, and 5.8% had FMD. Of the sample, 95% of our hypermobile patients with abnormal neuroimaging also had migraine. Our data verifies the previously documented very high prevalence rate of migraine in patients with HSD/hEDS [3,12].

Similarly to the current study, most patients seen at the Mayo Clinic EDS Clinic in prior studies received a diagnosis of HSD (66.5%) versus 20.3% for hEDS [4]. The majority of patients who were evaluated were White, non-Hispanic (HSD 92%, hEDS 92%), cis-females (HSD 93%, hEDS 88%) [4]. The excessive female predominant gender ratio noted in our present study reflects the HSD/hEDS populations and has been published previously by our group and others [2,3,20]. Although sex and gender are known to be important factors in HSD and hEDS, no studies have examined sex differences using large numbers of well-defined patients. Like HSD/hEDS, migraine occurs more often in women than men [21,22].

### 4.1. Unruptured Intracranial Aneurysms

Current documented risk factors for UIA include female sex, older age, history of smoking, hypertension, autosomal dominant polycystic kidney disease, and a family history of intracranial aneurysm or subarachnoid hemorrhage [23]. There is no definitive suggestion of hypermobility as a risk factor in previous studies, especially for the non-monogenetic subtypes. However, to date, very few studies have examined this issue, especially in patients with HSD/hEDS, the most common forms of EDS. The largest study (and really the only study) prior to our current investigation examined 99 patients with EDS and noted a prevalence of 12%, which is almost equal to our investigation, but most of the patients in that study with UIA had vEDS (7/12), while three had probable hEDS (HSD was not noted) [24]. That study was completed prior to the issuance of the 2017 EDS diagnostic criteria, and so the diagnoses may be categorized differently than in our study. The mean size of the aneurysms was about 7 mm, which is larger than what we found in our study, with most of these cases occurring in patients with vEDS. Aneurysm size was not documented per EDS subtype. Similarly to our study, most aneurysms in their investigation involved the internal carotid artery territory, and the mean age of the patients at the time of aneurysm diagnosis was 42 years. Age by specific EDS subtype was not defined. Data according to sex was also not documented for those with UIA, although most EDS patients were female (82%). In their study, 33% of the patients with intracranial aneurysms had headaches, but the exact headache subtype was not defined. Another study examined 448 EDS patients via a PubMed review spanning 60 years and found 135 head and neck aneurysms, of which 76% occurred in patients with vEDS, while only 2% were noted in hEDS (HSD was not documented) [25]. No other information was provided for EDS subtypes, such as aneurysm location or gender.

In our study, UIAs occurred in patients who were <50 years of age, with relatively small intracranial aneurysms in the anterior/carotid circulation, and a history of migraine (91%), possibly with aura. However, similar numbers of HSD and hEDS patients developed UIAs. Most aneurysm morphology was saccular. HSD patients mostly had episodic migraine, while hEDS patients had chronic migraine. The aneurysms were not considered to be part of a secondary headache etiology in any of the patients.

### 4.2. Spontaneous Cervical Artery Dissection

Patients with SCeAD often have signs of connective tissue abnormalities, and both Marfan syndrome and vEDS are known risk factors [5]. However, there is minimal data looking at the risk of SCeAD in patients with HSD and hEDS. In this study, we had more patients with a diagnosis of HSD (80% of our cohort) who were over 50 years of age at the time of SCeAD diagnosis, with vertebral artery predominance, and a history of migraine, which was noted in all SCeAD patients. The migraine subtype (episodic or chronic) and the presence of aura did not seem to influence the risk of SCeAD.

Previous studies suggest a lower prevalence of SCeAD in HSD and hEDS patients than we found in our cohort. A recent investigation of 258 patients with either HSD or hEDS (utilizing the 2017 EDS diagnostic criteria) reported SCeAD in only two patients: one HSD with both vertebral and carotid artery involvement and one hEDS with carotid artery involvement [26]. The sex of the patients was not documented. A multi-center study of 2201 individuals also found only two patients with hEDS and SCeAD [27]. A much larger study of over 9000 hospitalized patients with EDS noted an increased risk for cervical artery dissections (both carotid and vertebral) versus a non-EDS population; however, the subtypes of EDS studied were not documented, so it was not directly comparable to our population [28]. Notably, the number of patients with a primary headache diagnosis was not documented in any of those studies. Thus, if migraine or migraine plus hypermobility is necessary for developing SCeAD, that may explain the disparate results. A recent single-center study that examined sex differences in 144 patients with SCeAD found that slightly more females had a connective tissue disorder than males, but they did not define the type of connective tissue diagnosis [29]. Interestingly, the vertebral artery was more often involved in women in that study, similar to the possible preference for the vertebral artery in our female HSD patients, but diagnoses of HSD were not reported. The high prevalence of vertebral artery involvement in our SCeAD patients may indeed be unique to the hypermobile population versus the general population. However, this may just reflect the small number of positive cases from our small study population, and if more patients were studied, the typical carotid artery predominance would be noted [30].

### 4.3. Fibromuscular Dysplasia

In this study, we diagnosed FMD only utilizing intracranial and cervical artery imaging. Our patients with FMD had HSD, carotid artery involvement, were 50 years or older at the time of diagnosis, and had a history of migraine (which was noted in all patients with a chronic migraine predominance) and migraine with aura. There are only two prior studies examining the association between connective tissue disease and FMD, with disparate results. One study of 139 female FMD patients noted that the majority had Beighton scores of 2 or less, thus non-hypermobile, while only 2.9% were >5/9, indicating hypermobility [31]. Only 19% had more than four connective tissue physical characteristics, and most lacked signs of large joint hypermobility, although they did note a high prevalence of dental crowding, pectus issues, and palatal issues, which can be seen in hypermobile patients. A second study of 47 patients noted that 57% were hypermobile (positive Beighton score of 5 plus) and 96% had more than four clinical features associated with connective tissue syndromes [32]. Neither study used the current 2017 diagnostic criteria to define HSD or hEDS [6]. Finally, the largest study of FMD in the United States examined 4860 cases but did not mention hypermobility as part of the risk factor profile, and did not appear to have examined whether their patients were hypermobile or had HSD/hEDS [19]. This was a study of data obtained from the electronic medical record, and most physicians have not been trained about hypermobility, so these diagnoses are likely absent from the medical record [30].

### 4.4. Is It Hypermobility, Migraine, or Both?

#### 4.4.1. Unruptured Intracranial Aneurysm

There appears to be an increased risk of developing intracranial aneurysms in migraine sufferers, but the data is scant overall. Recently, a two-sample bidirectional Mendelian Randomization analysis utilizing genome-wide association data from an international headache group noted a possible increased risk for UIA in patients with migraine with aura but not without aura [33]. Migraine without aura was associated more with aneurysmal subarachnoid hemorrhage. Another study utilizing the same statistical technique but with a meta-analysis of five studies showed a possible increased risk in patients with a genetic predisposition to migraine (no specific migraine subtypes were documented), suggesting a potential causal relationship between the two conditions [34]. Another investigation utilizing data from two university centers noted a migraine prevalence of 24% in their patients with UIA [9]. This was higher than in their control population. The exact subtype of migraine the patients suffered from was not defined. Finally, another retrospective study showed a much higher migraine prevalence of 40% in patients with UIA or within a year of aneurysmal subarachnoid hemorrhage [35]. In this study, the majority had migraine without aura; however, 89% developed an aneurysmal rupture.

In none of these previously published investigations was a history of hypermobility noted. Thus, it was never considered to be a possible risk factor in patients with migraine who developed UIA. In our study, 91% of HSD/hEDS patients who developed UIAs had a migraine history (60% chronic migraine, 40% episodic migraine). As more than 50% of our patients also had a history of migraine with aura, and the fact that a recent study also suggested an aura connection in migraine patients with UIA, may indicate an increased risk of UIA in patients with HSD or hEDS and migraine with aura [33]. However, our data could also indicate an increased risk with hypermobility alone.

#### 4.4.2. Spontaneous Cervical Artery Dissections

There also appears to be a connection between a history of migraine and SCeAD. In our study, all HSD and hEDS patients with a dissection had migraine. There have been two meta-analysis studies looking at this association. The earlier investigation was relatively small, consisting of only five studies, which found a 2-fold increased risk of cervical artery dissection in migraineurs, with a stronger association with migraine without aura [36]. A more recent, larger study that examined 57 studies found that having a history of migraine was associated with 1.74-fold increased odds of SCeAD. The association again was primarily with migraine without aura [10]. In a single-center study looking at sex differences for SCeAD in 144 patients, significantly more females had a history of migraine, although migraine subtype was not defined [29]. In addition, slightly more women had the presence of a connective tissue disorder, although no specific connective tissue diagnosis was provided. Finally, a 19-year population-based study from Olmsted County, MN, noted 123 patients with cervical artery dissections, of which 37% had a history of migraine (with or without aura, and chronic vs. episodic migraine was not mentioned), with a slightly higher percentage of vertebral artery dissections in the migraine group [30]. None of these studies mentioned the number of patients with a history of concomitant hypermobility syndromes, again questioning the true role of migraine as a sole risk factor versus that of hypermobility. The authors from the Rochester Epidemiology Project study made the point that medical staff do not consistently document history or conduct an exam for connective tissue issues, thus they were unable to comment on their prevalence in their population [30]. As their migraine population had more vertebral artery dissections, it would be interesting to know how many had HSD, which we noted as increased in our population. As all of our patients had a history of migraine and most had migraine without aura, which appears to be the subtype of migraine noted in SCeAD patients (both our data and previously published studies), this could then suggest that having HSD/hEDS plus migraine without aura may act as co-risk factors for SCeAD development or it may just reflect hypermobility alone as the risk factor.

#### 4.4.3. Fibromuscular Dysplasia

FMD is a disorder without a defined etiology. It has been suggested that migraine is a potential risk factor for the development of FMD or at least has some pathophysiologic association. Upwards of 70% of FMD patients have a headache history, and migraine has been noted in 25–30% of patients [37]. In a large registry-based cohort of patients with FMD, approximately two-thirds experienced headaches (67.5%) [11]. Those with headaches were more likely to have extracranial carotid or vertebral artery FMD and be diagnosed at a younger age, 50.6 vs. 58.0 years, than those without headaches. Migraine-like headaches were noted in 52% of patients, although ICHD criteria were not utilized to define migraine subtype [15]. Chronic daily headache was noted in 27%, but the type of daily headache (chronic migraine, chronic tension type) was not documented. Recently, using a two-sample mediation Mendelian Randomization design and data from the International Headache Genetics Consortium, migraine was associated with FMD, although the type of migraine (with or without aura, episodic or chronic) was not mentioned [38]. No data on hypermobility was noted in the study.

Our investigation is unique in that we examined FMD in a defined headache population with a defined hypermobility diagnosis. We found that all patients with FMD had a history of migraine and that individuals with HSD, chronic migraine, and a history of aura were at the highest risk, with the carotid arteries preferentially affected compared to the vertebral arteries. In the one other study examining the connection between connective tissue disorders and FMD, there was a headache prevalence of 53% in patients who showed signs of hypermobility, but as they did not define a specific hypermobile subtype and no primary headache diagnosis was documented, it was not feasible to glean possible risk factor information from that study [32]. Our study suggests that having HSD/hEDS and a history of migraine with aura may act as co-risk factors for the presence of FMD of the head and neck vessels, but it could also just reflect hypermobility as a sole risk factor.

### 4.5. Neuroimaging Screening

At present, there are no neuroimaging screening guidelines for patients with hEDS and HSD. Surprisingly, very few of our patients had any previous neurovascular imaging prior to their headache consultation; however, the majority were not diagnosed with a hypermobility syndrome until they were evaluated at our center. Our data suggests the need for at least targeted screening for cerebral/cervical arterial issues in HSD/hEDS patients who present with a complaint of headache or migraine (either episodic or chronic). We believe that our patients had primary migraine and that the vessel abnormalities were incidental imaging findings. This was based on the onset of their ICHD-defined primary headaches at a younger age (pre-teens, teens, early twenties) and typically years prior to their positive neuroimaging results. The need for universal screening for all patients with hEDS and HSD can be considered based on our findings, but for true cost-effectiveness, larger-scale studies of these populations are warranted. In most instances, aside from SCeAD, which can truly alter subsequent lifestyle choices (i.e., no rollercoasters, chiropractic manipulation, etc.), the abnormalities we identified were not considered to be symptomatic, rarely needing intervention or altering treatment. However, identifying individuals with long-term risk for aneurysm rupture, re-dissection with activity, and having an elevated risk of stroke from FMD are very important collateral findings from neuroimaging.

### 4.6. Limitations

Our study has several limitations. First, this is a cohort study reporting findings from patients being referred to tertiary specialty clinics, which introduces a selection bias. This cohort is predominantly White, non-Hispanic females and so may not be generalizable to other races, ethnicities, or sexes. As most of our patients were female, we cannot make any trend statements for vessel abnormalities in our population based on gender. We can suggest that having hypermobility alone is sufficient to develop neurovascular abnormalities, but without a larger study population or true control group, we cannot make any conclusions on the added role of migraine as a possible co-risk factor or independent risk factor. In addition, we did not specifically look at all potential risk factors for UIA, SCeAD, or FMD in this study. A prior smoking history, however, was only noted in one of our neuroimaging positive patients, so it was not a causative link. Regarding family history, we asked only about intracranial aneurysms but did not inquire about familial SCeAD or FMD. The lack of family history of UIA in our cohort would further the argument for the need for neurovascular imaging in this patient population based on our prevalence figures. In our study, FMD was diagnosed only with imaging of the head and neck vessels. Full cross-sectional imaging of the chest, abdomen, and pelvis, which potentially could have identified additional cases of FMD and possibly other arterial complications, was not conducted. Thus, we can only suggest an association of HSD/hEDS with FMD and cervical arteriopathy and not FMD with systemic arterial issues. Could there be unique subsets of FMD in which patients with hypermobility and possibly concomitant migraine present differently than those without these conditions? It is feasible that some of our patients may have had underlying genetic mutations that were not screened for. Of note, all our imaging-positive patients were seen by neurovascular specialists, and only one patient was sent for genetic consultation; genetic screening was negative for underlying monogenetic connective tissue disorders. In addition, we did not have a true control population per se; however, we did identify 13 patients of which 12 were White, non-Hispanic women, with a mean average age of 41 years and headache diagnoses: three episodic migraine, seven chronic migraine, two with a history of aura, two NDPH, and one chronic tension-type headache who were not found to have a hypermobility diagnosis by the EDS Clinic but had the appropriate neurovascular imaging. None of these patients developed UIA, SCeAD, or FMD, thus possibly strengthening our positive results and the role of hypermobility alone and not migraine as a risk factor in neurovascular anomaly development. Finally, the number of cases in this study was small, and thus larger population-based studies are needed to confirm our findings. However, this study is the largest to date to examine vessel abnormalities in patients with HSD or hEDS using the 2017 diagnostic criteria, who also have headache/migraine defined with ICHD criteria at an academic headache center. Our increased prevalence findings indicate that further research is needed and suggest that patients with HSD/hEDS and possibly migraine should be evaluated for cerebral/cervical vessel abnormalities on clinical exam and neuroimaging.

## 5. Conclusions

This is the first study to identify that patients with the more common type of EDS, HSD and hEDS, and possibly a concomitant history of migraine, have a heightened risk for the development of UIA, SCeAD, and FMD. Our findings suggest the need for targeted screening with intracranial and extracranial arterial imaging for this unique patient population, as these vessel abnormalities may lead to patient morbidity if missed. Large-scale studies across multiple centers will be needed to verify our findings.

## Figures and Tables

**Table 1:** Demographics (*n* = 103).

Characteristic	HSD *n* = 72	hEDS *n* = 31	*p* Value ^α^
*Sex, n (%)*			0.99
Female	69 (95.8%)	30 (96.8%)	
Male	3 (4.2%)	1 (3.2%)	
*Age, mean, range in years*	37, 18–67	38, 20–55	0.97
*Race, n (%)*			
American Indian/Alaskan Native	0 (0%)	0 (0%)	-
Asian	1 (1.4%)	0 (0%)	0.99
Black or African American	2 (2.8%)	0 (0%)	0.99
Native Hawaiian/Pacific Islander	0 (0%)	0 (0%)	-
White	65 (90.3%)	31 (100.0%)	0.10
Other	0 (0%)	0 (0%)	-
Unknown/Not Disclosed	4 (5.6%)	0 (0%)	0.31
*Ethnicity, n (%)*			
Hispanic	10 (13.9%)	2 (6.5%)	0.34
Not Hispanic	59 (81.9%)	29 (93.5%)	0.22
Unknown/Not Disclosed	3 (4.2%)	0 (0%)	0.55
*Headache History, n (%)*			
Migraine	63 (87.5%)	28 (90.3%)	0.99
Episodic Migraine	37 (51.4%)	17 (54.8%)	0.83
Chronic Migraine	26 (36.1%)	11 (35.5%)	0.99
New Daily Persistent Headache	9 (12.5%)	3 (9.7%)	0.99

Abbreviations: HSD—hypermobile spectrum disorder, hEDS—hypermobile Ehlers–Danlos syndrome. ^α^ *p*-values obtained from Fisher’s exact test for categorical data.

**Table 2:** Vascular defects in hypermobile patients with headache/migraine (*n* = 103).

Vessel Abnormality	HSD *n =* 72	hEDS *n* = 31	*p* Value *^a^*
Unruptured intracranial aneurysm (UIA)	6	5	0.30
Spontaneous cervical artery dissection (SCeAD)	4	1	1.00
Fibromuscular dysplasia (FMD)	5	1	0.67

HSD—hypermobile spectrum disorders; hEDS—hypermobile Ehlers–Danlos syndrome. *^a^* *p*-values obtained from Fisher’s exact test for categorical data.

## Data Availability

The original contributions presented in this study are included in the article. Further inquiries can be directed to the corresponding author.

## References

[1] Tinkle B., Castori M., Berglund B., Cohen H., Grahame R., Kazkaz H., Levy H. (2017). Hypermobile Ehlers-Danlos syndrome (a.k.a. Ehlers-Danlos syndrome Type III and Ehlers-Danlos syndrome hypermobility type): Clinical description and natural history. Am. J. Med. Genet. C Semin. Med. Genet..

[2] Castori M., Camerota F., Celletti C., Grammatico P., Padua L. (2010). Ehlers-Danlos syndrome hypermobility type and the excess of affected females: Possible mechanisms and perspectives. Am. J. Med. Genet. A.

[3] Fairweather D., Bruno K.A., Darakjian A.A., Bruce B.K., Gehin J.M., Kotha A., Jain A., Peng Z., Hodge D.O., Rozen T.D. (2023). High Overlap in Patients Diagnosed with Hypermobile Ehlers-Danlos Syndrome or Hypermobile Spectrum Disorders with Fibromyalgia and 40 Self-Reported Symptoms and Comorbidities. Front. Med..

[4] Darakjian A.A., Bhutani M., Fairweather D., Kocsis S.C., Fliess J.J., Khatib S., Weigel G.J., McCabe E.J., Balamurugan V., Perona E.E. (2024). Similarities and differences in self-reported symptoms and comorbidities between hypermobile Ehlers-Danlos syndrome and hypermobility spectrum disorders. Rheumatol. Adv. Pract..

[5] Kim S.T., Brinjikji W., Lanzino G., Kallmes D.F. (2016). Neurovascular manifestations of connective-tissue diseases: A review. Interv. Neuroradiol..

[6] Malfait F., Francomano C., Byers P., Belmont J., Berglund B., Black J., Bloom L., Bowen J.M., Brady A.F., Burrows N.P. (2017). The 2017 international classification of the Ehlers-Danlos syndromes. Am. J. Med. Genet. C Semin. Med. Genet..

[7] Knight D.R., Bruno K.A., Singh A., Munipalli B., Gajarawala S., Solomon M., Kocsis S.C., Darakjian A.A., Jain A., Whelan E.R. (2024). Cardiac defects of hypermobile Ehlers-Danlos syndrome and hypermobility spectrum disorders: A retrospective cohort study. Front. Cardiovasc. Med..

[8] Dong L., Dong W., Jin Y., Jiang Y., Li Z., Yu D. (2025). The Global Burden of Migraine: A 30-Year Trend Review and Future Projections by Age, Sex, Country, and Region. Pain Ther..

[9] Witvoet E.H., Pelzer N., Terwindt G.M., Rinkel G.J., Vlak M.H., Algra A., Wermer M.J. (2017). Migraine prevalence in patients with unruptured intracranial aneurysms: A case-control study. Brain Behav..

[10] Sun Z., Kleine-Borgmann J., Suh J., McDermott G.C., Vishnevetsky A., Rist P.M. (2023). Migraine and the risk of cervical artery dissection: A systematic review and meta-analysis. Eur. Stroke J..

[11] Wells B.J., Modi R.D., Gu X., Bumpus S.M., Swan K., Froehlich J.B., Gray B.H., Southerland A.M., Kim E.S., Fendrikova Mahlay N. (2020). Clinical associations of headaches among patients with fibromuscular dysplasia: A Report from the US Registry for Fibromuscular Dysplasia. Vasc. Med..

[12] Mehta D., Simmonds L., Hakim A.J., Matharu M. (2024). Headache disorders in patients with Ehlers-Danlos syndromes and hypermobility spectrum disorders. Front. Neurol..

[13] Knight D.R., Confiado S.M., Bruno K.A., Fairweather D., Seymour-Sonnier A.M., Jain A., Gehin J.M., Whelan E.R., Culberson J.H., Munipalli B. (2022). Establishing an Ehlers-Danlos Syndrome Clinic: Lessons Learned. SN Compr. Clin. Med..

[14] Morlino S., Castori M. (2023). Placing joint hypermobility in context: Traits, disorders and syndromes. Br. Med. Bull..

[15] Fairweather D., Bruno K.A., Darakjian A.A., Wilson F.C., Fliess J.J., Murphy E.F., Kocsis S.C., Strandes M.W., Weigel G.J., Puls A.M. (2025). Localized and historical hypermobility spectrum disorders share self-reported symptoms and comorbidities with hEDS and HSD. Front. Med..

[16] Headache Classification Committee of the International Headache Society (IHS) (2018). The International Classification of Headache Disorders, 3rd edition. Cephalalgia.

[17] Brott T.G., Halperin J.L., Abbara S., Bacharach J.M., Barr J.D., Bush R.L., Cates C.U., Creager M.A., Fowler S.B., Friday G. (2011). ASA/ACCF/AHA/AANN/AANS/ACR/ASNR/CNS/SAIP/SCAI/SIR/SNIS/SVM/SVS guideline on the management of patients with extracranial carotid and vertebral artery disease: A report of the American College of Cardiology Foundation/American Heart Association Task Force on Practice Guidelines, and the American Stroke Association, American Association of Neuroscience Nurses, American Association of Neurological Surgeons, American College of Radiology, American Society of Neuroradiology, Congress of Neurological Surgeons, Society of Atherosclerosis Imaging and Prevention, Society for Cardiovascular Angiography and Interventions, Society of Interventional Radiology, Society of NeuroInterventional Surgery, Society for Vascular Medicine, and Society for Vascular Surgery. J. Am. Coll. Cardiol..

[18] Chengazi H.U., Bhatt A.A. (2019). Pathology of the carotid space. Insights Imaging.

[19] Gornik H.L., Persu A., Adlam D., Aparicio L.S., Azizi M., Boulanger M., Bruno R.M., De Leeuw P., Fendrikova-Mahlay N., Froehlich J. (2019). First International Consensus on the diagnosis and management of fibromuscular dysplasia. Vasc. Med..

[20] Rodgers K.R., Gui J., Dinulos M.B., Chou R.C. (2017). Ehlers-Danlos syndrome hypermobility type is associated with rheumatic diseases. Sci. Rep..

[21] Burch R., Rizzoli P., Loder E. (2021). The prevalence and impact of migraine and severe headache in the United States: Updated age, sex, and socioeconomic-specific estimates from government health surveys. Headache.

[22] Diamond S., Bigal M.E., Silberstein S., Loder E., Reed M., Lipton R.B. (2007). Patterns of diagnosis and acute and preventive treatment for migraine in the United States: Results from the American Migraine Prevalence and Prevention study. Headache.

[23] Brown R.D., Broderick J.P. (2014). Unruptured intracranial aneurysms: Epidemiology, natural history, management options, and familial screening. Lancet Neurol..

[24] Kim S.T., Brinjikji W., Kallmes D.F. (2016). Prevalence of Intracranial Aneurysms in Patients with Connective Tissue Diseases: A Retrospective Study. Am. J. Neuroradiol..

[25] Shabani M., Abdollahi A., Brar B.K., MacCarrick G.L., Ambale Venkatesh B., Lima J.A., Bodurtha J.N. (2023). Vascular aneurysms in Ehlers-Danlos syndrome subtypes: A systematic review. Clin. Genet..

[26] Rashed E.R., Ruiz Maya T., Black J., Fettig V., Kadian-Dodov D., Olin J.W., Mehta L., Gelb B.D., Kontorovich A.R. (2022). Cardiovascular manifestations of hypermobile Ehlers-Danlos syndrome and hypermobility spectrum disorders. Vasc. Med..

[27] Debette S., Goeggel Simonetti B., Schilling S., Martin J.J., Kloss M., Sarikaya H., Hausser I., Engelter S., Metso T.M., Pezzini A. (2014). Familial occurrence and heritable connective tissue disorders in cervical artery dissection. Neurology.

[28] Kim S.T., Cloft H., Flemming K.D., Kallmes D.F., Lanzino G., Brinjikji W. (2017). Increased Prevalence of Cerebrovascular Disease in Hospitalized Patients with Ehlers-Danlos Syndrome. J. Stroke Cerebrovasc. Dis..

[29] Schipani E., Griffin K.J., Oakley C.I., Keser Z. (2025). Sex differences in the epidemiology of spontaneous and traumatic cervical artery dissections. Stroke Vasc. Neurol..

[30] Griffin K.J., Harmsen W.S., Mandrekar J., Brown R.D., Keser Z. (2024). Epidemiology of Spontaneous Cervical Artery Dissection: Population-Based Study. Stroke.

[31] O’Connor S., Kim E.S., Brinza E., Moran R., Fendrikova-Mahlay N., Wolski K., Gornik H.L. (2015). Systemic connective tissue features in women with fibromuscular dysplasia. Vasc. Med..

[32] Ganesh S.K., Morissette R., Xu Z., Schoenhoff F., Griswold B.F., Yang J., Tong L., Yang M.L., Hunker K., Sloper L. (2014). Clinical and biochemical profiles suggest fibromuscular dysplasia is a systemic disease with altered TGF-β expression and connective tissue features. FASEB J..

[33] Ren C., Gao Q., Li X., Yang F., Wang J., Guo P., Duan Z., Kong Y., Bi M., Chen L. (2025). Headache and Intracranial Aneurysm: A Bidirectional Mendelian Randomization Study. Curr. Neurovasc. Res..

[34] Daghlas I., Rist P.M., Chasman D.I. (2025). Genetically proxied liability to migraine and risk of intracranial aneurysm and subarachnoid hemorrhage. Headache.

[35] Lebedeva E.R., Gurary N.M., Sakovich V.P., Olesen J. (2013). Migraine before rupture of intracranial aneurysms. J. Headache Pain.

[36] Rist P.M., Diener H.C., Kurth T., Schürks M. (2011). Migraine, migraine aura, and cervical artery dissection: A systematic review and meta-analysis. Cephalalgia.

[37] O’Connor S.C., Poria N., Gornik H.L. (2015). Fibromuscular dysplasia: An update for the headache clinician. Headache.

[38] Chen Y.H., Yan F. (2025). Effect of genetic liability to migraines on spontaneous coronary artery dissection and fibromuscular dysplasia. Am. J. Med. Sci..

